# Mutational profiles in triple-negative breast cancer defined by ultradeep multigene sequencing show high rates of PI3K pathway alterations and clinically relevant entity subgroup specific differences

**DOI:** 10.18632/oncotarget.2481

**Published:** 2014-09-16

**Authors:** Mark Kriegsmann, Volker Endris, Thomas Wolf, Nicole Pfarr, Albrecht Stenzinger, Sibylle Loibl, Carsten Denkert, Andreas Schneeweiss, Jan Budczies, Peter Sinn, Wilko Weichert

**Affiliations:** ^1^ Institute of Pathology, University of Heidelberg, Germany; ^2^ German Cancer Research Center, Heidelberg, Germany; ^3^ German Breast Group, Neu-Isenburg, Germany; ^4^ Institute of Pathology, University Hospital Charité Berlin, Germany; ^5^ National Center for Tumor Diseases, Heidelberg, Germany; ^6^ German Cancer Consortium (DKTK), Germany

**Keywords:** triple-negative breast cancer, next generation sequencing, immunohistochemistry, mutation profiling

## Abstract

Mutational profiling of triple-negative breast cancer (TNBC) by whole exome sequencing (WES) yielded a landscape of genomic alterations in this tumor entity. However, the clinical significance of these findings remains enigmatic. Further, integration of WES in routine diagnostics using formalin-fixed paraffin-embedded (FFPE) material is currently not feasible.

Therefore, we designed and validated a breast cancer specific gene panel for semiconductor-based sequencing comprising 137 amplicons covering mutational hotspots in 44 genes and applied this panel on a cohort of 104 well-characterized FFPE TNBC with complete clinical follow-up.

*TP53* mutations were present in more than 80% of cases. PI3K pathway alterations (29.8%) comprising mainly *PIK3CA* mutations (22.1%) but also mutations and/or amplifications/deletions in other PI3K-associated genes (7.7%) were far more frequently observed, when compared to WES data. Alterations in MAPK signaling genes (8.7%) and cell-cycle regulators (14.4%) were also frequent. Mutational profiles were linked to TNBC subgroups defined by morphology and immunohistochemistry. Alterations in cell-cycle pathway regulators were linked with better overall (p=0.053) but not disease free survival.

Taken together, we could demonstrate that breast cancer targeted hotspot sequencing is feasible in a routine setting and yields reliable and clinically meaningful results. Mutational spectra were linked to clinical and immunohistochemically defined parameters.

## INTRODUCTION

Breast cancer is the most common malignant tumor in females and ranks first among cancer related deaths in woman worldwide, with estimated 508.000 deaths reported for 2011 [1]. In the last decade new molecular methods have substantially increased our knowledge of breast cancer biology with the ultimate promise to expand and improve current treatment options. The molecular era of breast cancer characterization started with gene expression studies. Gene expression based molecular breast cancer classification has first been introduced in 2000 [2] and later been validated [3]. In the following, it became clear that breast cancer is not one single entity but rather encompasses distinct intrinsic subtypes. These subtypes differ substantially in genomic complexity and driver alterations with tremendous impact on treatment response and clinical prognosis [3]. Despite these molecular stratification efforts, in routine diagnostics, immunohistochemical analyses are still the gold standard to determine the clinically relevant subtypes of breast cancer, namely luminal A, luminal B, HER-2 and triple-negative. While luminal and HER-2 subtypes may be eligible for targeted therapy [4, 5], patients with triple-negative breast cancer (TNBC) quite homogenously receive conventional chemotherapy, despite the fact that even the TNBC subgroup cannot be regarded as a single entity but rather as a trunk of heterogeneous diseases [6]. This is supported by gene expression studies that revealed further prognostic and predictive subgroups in TNBC. Besides molecular methods [3, 7-11], immunohistochemistry, as an easy to use readily available diagnostic tool, has also been used to delineate different subtypes of TNBC. Using a comprehensive immunohistochemical approach combined with hierarchical clustering, we have previously developed an algorithm to define four molecular subgroups of TNBC with distinct histopathological features and prognostic impact: luminal, basoluminal, basal A and basal B [12].

Genomic profiles of TNBC have recently been defined using whole exome sequencing. The respective studies revealed that TNBC patients harbor germ line mutations in tumor relevant genes [13] but also high numbers of somatic mutations [14-17]. Although these current genetic profiling studies have significantly expanded our molecular view on TNBC, they have some limitations as of sample size, clinical annotations and whole exome sequencing (WES) read depth. Moreover, WES, as the preferred method used within these studies, is expensive, time consuming and for a multitude of reasons cannot readily be implemented into large scale routine diagnostics yet [18].

Therefore, to translate the application of next-generation sequencing technologies into the clinical routine setting targeted multigene sequencing approaches, either amplicon or capture based, have been developed [18]. In this regard several commercially available pan-cancer panels press into the market, however, such one-size-fits-all panels comprise a multitude of genes that are rarely or never mutated in breast cancer and are not satisfactory with respect to a sufficient cost-to-information ratio. Therefore, we have recently started to develop and validate entity specific, amplicon-based gene panels for next-generation ultradeep parallel multigene sequencing on IonTorrent devices. With this approach that has been implemented in the routine pathology diagnostics pipeline at our institution recently, we are able to generate clinically relevant, valid molecular stratification data in a time and cost effective manner [19].

In the present study, we created and validated a breast cancer specific gene panel and applied it to a cohort of 104 TNBC cases in order to decipher the genetic landscape of these tumors. We compared our results with published mutational profiles from exome data to check for the validity of our breast cancer specific targeted multigene sequencing approach. Further, we sought to identify specific mutational patterns with prognostic potential and we investigated whether the immunohistochemical stratification approach previously developed by us [12] can be correlated with specific mutational profiles of TNBC.

## RESULTS

### Altered pathways

**Figure 1:**
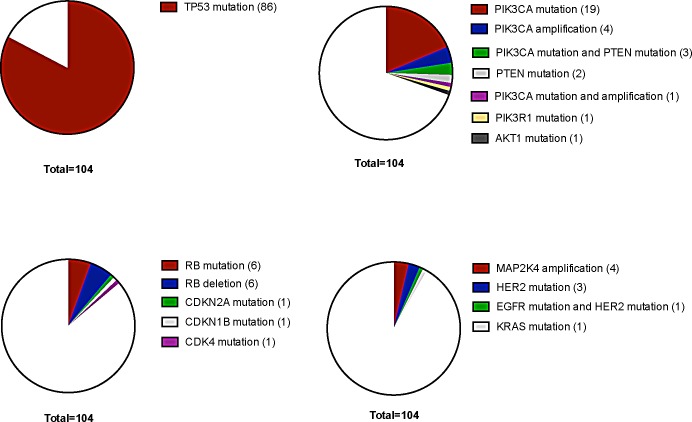
Distribution of molecular alterations sorted for molecularly defined subtypes in TNBC

The most frequently mutated gene in our TNBC cohort was *TP53* with a mutational frequency of 82.7% (86 out of 104 cases) (Figure 1). Of these, only 14 cases (16.3%) showed an allele frequency indicative of subclonality, as arbitrarily defined by a threshold of below 40%.

An overall of 31 out of 104 tumors (29.8%) showed genetic alterations in at least one gene implicated in PI3K signaling (Figure 1). In this pathway, *PIK3CA* was most frequently affected (23 out of 104 tumors (22.1%)). For *PIK3CA,* putatively subclonal allele frequencies were much more frequent than for *TP53* with 10 cases (43.5%) showing an allele frequency of below 40% (Figure 3).

A third set of genes, which was frequently altered belonged to a category of cell cycle regulators other than *TP53*. 15 out of 104 tumors (14.4%) had at least one genomic aberration in cell cycle associated pathways, here *RB* was the most frequently affected gene with 6 point mutations and 6 deletions (Figure 1).

**Table 1:** Frequency of molecular subgroups and correlation with clinicopathological parameters

			TP53 mutations			PI3K pathway alterations			Cell cycle alterations			MAPK pathway alterations		
		*overall*	*wildtype*	*mutated*	*p-value*	*not altered*	*altered*	*p-value*	*not altered*	*altered*	*p-value*	*not altered*	*altered*	*p-value*
		104 (100%)	18 (17.3%)	86 (82.7%)		73 (70.2%)	31 (29.8%)		89 (85.6%)	15 (14.4%)		95 (91.3%)	9 (8.7%)	
Age														
	*mean*	54.1	64.3	52.0	***0.001***	52.0	59.2	***0.020***	53.2	60.1	***0.033***	53.8	58.2	*0.383*
pT														
	*pT1*	39 (37.5%)	8 (20.5%)	31 (79.5%)	*0.845*	29 (74.4%)	10 (25.6%)	*0.936*	34 (87.2%)	5 (12.8%)	*0.635*	34 (87.2%)	5 (12.8%)	*0.694*
	*pT2*	52 (50%)	7 (13.5%)	45 (86.5%)		33 (63.5%)	19 (36.5%)		43 (82.7%)	9 (17.3%)		50 (96.2%)	2 (3.8%)	
	*pT3/4*	13 (12.5%)	3 (23.1%)	10 (76.9%)		11 (84.6%)	2 (15.4%)		12 (92.3%)	1 (7.7%)		11 (84.6%)	2 (15.4%)	
pN*														
	*pN0*	61 (60.4%)	12 (19.7%)	49 (80.3%)	*0.689*	44 (72.1%)	17 (27.9%)	*0.890*	49 (80.3%)	12 (19.7%)	*0.597*	57 (93.4%)	4 (6.6%)	*0.438*
	*pN1*	22 (21.8%)	2 (9.1%)	20 (90.9%)		15 (68.2%)	7 (31.8%)		22 (100%)	0 (0%)		20 (90.9%)	2 (9.1%)	
	*pN2*	10 (9.9%)	2 (20%)	8 (80%)		4 (40%)	6 (60%)		9 (90%)	1 (10%)		7 (70%)	3 (30%)	
	*pN3*	8 (7.9%)	1 (12.5%)	7 (87.5%)		8 (100%)	0 (0%)		6 (75%)	2 (25%)		8 (100%)	0 (0%)	
Stage														
	*I*	29 (27.9%)	7 (24.1%)	22 (75.9%)	*0.753*	22 (75.9%)	7 (24.1%)	*0.543*	25 (86.2%)	4 (13.8%)	*0.752*	26 (89.7%)	3 (10.3%)	*0.672*
	*II*	52 (50%)	6 (11.5%)	46 (88.5%)		35 (67.3%)	17 (32.7%)		44 (84.6%)	8 (15.4%)		49 (94.2%)	3 (5.8%)	
	*III*	17 (16.3%)	2 (11.8%)	15 (88.2%)		13 (76.5%)	4 (23.5%)		14 (82.4%)	3 (17.6%)		15 (88.2%)	2 (11.8%)	
	*IV*	6 (5.8%)	3 (50%)	3 (50%)		3 (50%)	3 (50%)		6 (100%)	0 (0%)		5 (83.3%)	1 (16.7%)	
Grade														
	*G2*	6 (5.8%)	2 (33.3%)	4 (66.7%)	*0.277*	4 (66.7%)	2 (33.3%)	*1.000*	4 (66.7%)	2 (33.3%)	*0.207*	6 (100%)	0 (0%)	*1.000*
	*G3*	98 (94.2%)	16 (16.3%)	82 (83.7%)		69 (70.4%)	29 (29.6%)		85 (86.7%)	13 (13.3%)		89 (90.8%)	9 (9.2%)	
Margin*														
	*pushing*	43 (48.3%)	5 (11.6%)	38 (88.4%)	*0.869*	33 (76.7%)	10 (23.3%)	*0.077*	36 (83.7%)	7 (16.3%)	*0.548*	42 (97.7%)	1 (2.3%)	*0.165*
	*infiltrative*	25 (28.1%)	7 (28%)	18 (72%)		13 (52%)	12 (48%)		23 (92%)	2 (8%)		22 (88%)	3 (12%)	
	*mixed*	21 (23.6%)	2 (9.5%)	19 (90.5%)		16 (76.2%)	5 (23.8%)		19 (90.5%)	2 (9.5%)		18 (85.7%)	3 (14.3%)	
Inflammation*														
	*scarce*	16 (17.6%)	5 (31.3%)	11 (68.7%)	*0.099*	12 (75%)	4 (25%)	*0.680*	12 (75%)	4 (25%)	*0.146*	15 (93.7%)	1 (6.3%)	*0.875*
	*moderate*	24 (26.4%)	3 (12.5%)	21 (87.5%)		14 (58.3%)	10 (41.7%)		21 (87.5%)	3 (12.5%)		22 (91.7%)	2 (8.3%)	
	*marked*	51 (56%)	6 (11.8%)	45 (88.2%)		38 (74.5%)	13 (25.5%)		46 (90.2%)	5 (9.8%)		47 (92.2%)	4 (7.8%)	
Ki-67														
	*mean*	57.0	48.3	58.9	*0.163*	62.6	44.1	***0.001***	57.1	56.3	*0.923*	59.2	34.4	***0.014***

* data on N status, margin and inflammation was missing for 3, 15 and 13 cases, respectively.

**Figure 2:**
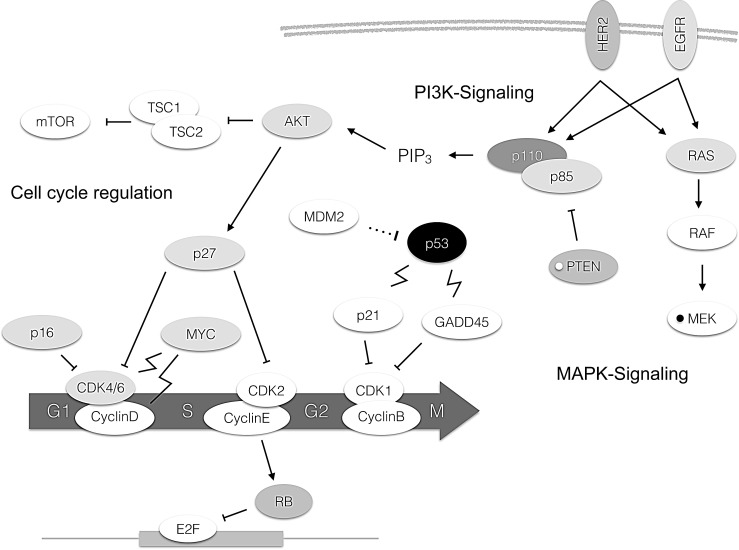
Molecular alterations in TNBC depicted in the pathway context The darkness of the boxes indicates frequency of mutations of the respective gene. A white small circle within a box indicates deletions, a black small circle amplifications. Arrow: Activation. Bar: Inhibition. Dotted line: Degradation. Flash: Transcriptional upregulation.

The fourth set of genes found to be altered in a relevant fraction of tumors affected MAPK signaling. 9 out of 104 (8.7%) cases of TNBC had alterations in this pathway with *MAP2K4* amplifications (4 cases) and *HER2* mutations (4 cases) being the most frequent events. Genetic aberrations in all other genes included in our cancer specific panel were of low frequency and did not cluster in a certain pathway. This includes cases with mutations in *TBX3, CDH1, HERC1, RPS6KA1, PTPRD, GATA3* and *MYC* (each gene mutated in one case with the exception of CDH1 which was found mutated in 3 cases and PTPRD which was mutated in two cases). Overall, 76 cases (73%) had one coding mutation, 21 cases (20.2%) had two mutations, six cases harbored three mutations (5.8%) and one case (1%) had four coding mutations as could be detected by our panel. A map showing frequently altered genes and their interaction with each other is shown in Figure 2.

### Overlap of pathway alterations

**Figure 3:**
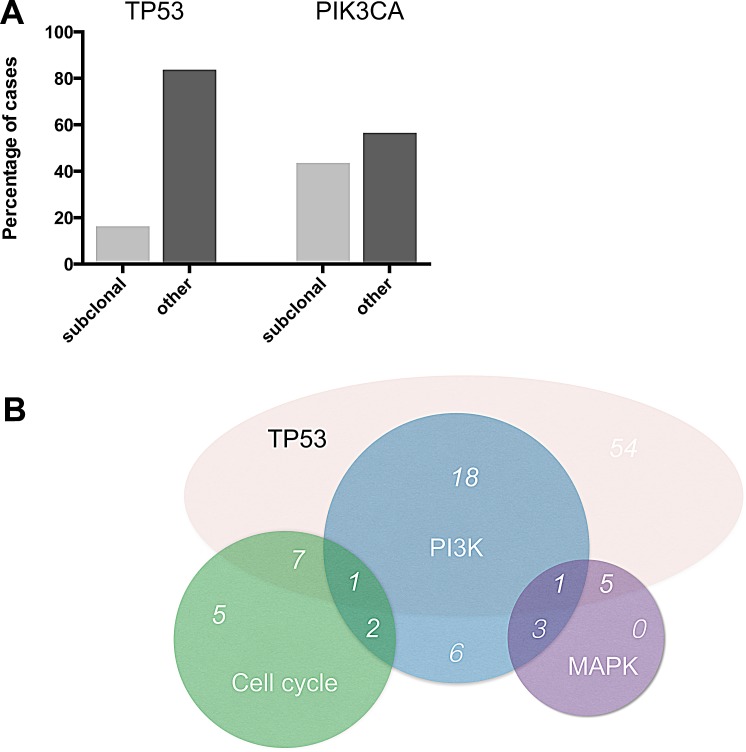
Subclonality and overlap of pathway alterations (A) Percentage of events with allele frequencies below 40% for the two most frequently mutated TNBC genes *TP53* and *PIK3CA*. (B) Overlap of pathway alterations.

20 out of 31 (64.5%) cases with PI3K pathway alterations also harbored mutations in *TP53*. A comparable overlap rate was seen for MAPK pathway alterations and the presence of *TP53* mutations (6 out of 9 cases, 66.7%). The combination of genomic alterations was less frequent for genes encoding cell cycle proteins and *TP53*, with just over 50% of the cases (8 out of 15 cases, 53.3%) with cell cycle aberrations having a concomitant *TP53* mutation. 44.9% of TNBC cases with MAPK pathway alterations also had altered genes in the PI3K pathway (4 out of 9 cases) while this was only observed in 20% of cases with aberrations in cell cycle pathways (3 out of 15 cases) (Figure 3). We did not identify cases with concomitant cell cycle and MAPK pathway alterations. Overall, the presence of two key genetic pathway alterations was more frequent than the presence of just one altered driver pathway.

### Mutational profiles in immunohistochemically defined subgroups

**Figure 4:**
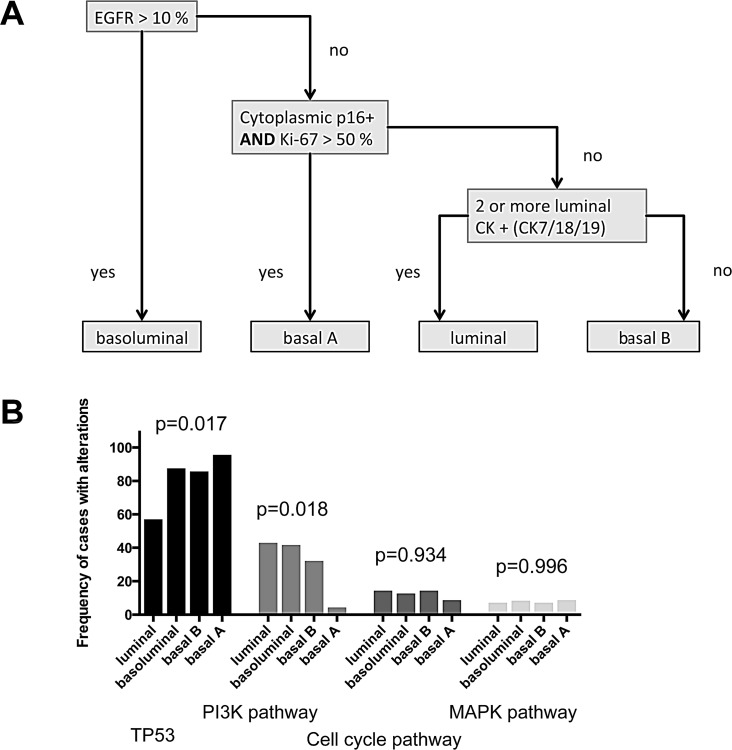
Immunohistochmically defined subgoups and molecular profiles (A) Immunohistochemical classification algorithm (according to Elsawaf et al. [12]) (B) Distribution of pathway alterations according to IHC TNBC subgroups.

Previously, we defined highly prognostic subgroups of TNBC, which can be delineated by immunohistochemistry [12] into a luminal-like, a basoluminal, and a basal B as well as a basal A TNBC subtype (Figure 4). Data on both, genetic and immunohistochemical profiles were present for 89 cases of our cohort. When correlating our mutational profiles with subgroups defined by immunohistochemistry we found some striking associations. While only 57.1% of luminal-like TNBC cases (n=14) had *TP53* mutations, frequencies were considerably higher in the other subgroups with 87.5% of basoluminal (n=24), 85.7% of basal B (n=28) and 95.7% (n=22) of basal A cases being positive (p=0.017). p53 protein expression in itself was tightly linked to the presence of *TP53* mutations. *TP53* wildtype cases showed a median of 0% p53 expressing tumor cells, while in the *TP53* mutant group the median number of p53 positive cells was 60% (p<0.001, data not shown).

PI3K alterations, when compared to the data for *TP53* mutations, showed the opposite trend in the association with IHC defined subgroups with 42.9% of luminal-like, 41.7% of basoluminal, 34.6% of basal B and only 4.3% of basal A cases being altered (p=0.018). Cell cycle alterations and MAPK pathway aberrations were not enriched in one of the subgroups defined by immunohistochemistry.

### Mutational profiles and clinical data

*TP53* mutations were frequent in invasive carcinomas NST (73 out of 88 cases, 84.9%) and in medullary carcinomas (MED: 5 out of 5 cases, 100%) as well as in the rare subtypes (carcinoma with apocrine differentiation and metaplastic carcinoma) (4 out of 4 cases, 100%) but considerably less frequent in invasive lobular carcinomas (ILC: 4 out of 7 cases, 63.6%). PI3K pathway alterations were also frequent in invasive carcinomas NST (28 out of 88 cases, 31.8%), while such events were rare in ILC (1 out of 7 cases, 14.3%) and MED (1 out of 5 cases, 20%). 1 out of 4 cases of the rare subtypes was found positive (25%) for PI3K pathway alterations. Genetic alterations in cell cycle genes were only found in invasive carcinomas NST (13 out of 88 cases, 14.8%) and in invasive lobular carcinoma (2 out of 7 cases, 28.6%) but not in any of the more rare subtypes. MAPK alterations were exclusively observed in invasive carcinomas NST (9 out of 88 cases, 10.2%). Age at time of diagnosis was significantly linked with mutational profiles, patients with *TP53* driven tumors were significantly younger, while patients with PI3K and cell cycle alterations were significantly older (Table 1).

Neither the presence of *TP53* mutations, nor of PI3K, cell cycle or MAPK pathway alterations showed any association with local tumor extent (pT), nodal spread (pN) or with tumor grade (Table 1).

### Mutational profiles and tumor properties

Although none of the molecular groups was significantly linked to the configuration of resection margin or inflammatory antitumor reaction in TNBC, our data suggest that PI3K driven tumors tended towards a more infiltrative phenotype (p=0.077), while *TP53*-driven neoplasms were by trend associated with stronger inflammation (p=0.099)(Table 1).

Proliferative capacity was significantly linked to mutational profiles. Tumors with PI3K mutations as well as tumors with MAPK pathway activations were associated with lower proliferation levels (p=0.001 and p=0.014, respectively).

### Mutational profiles and survival

**Figure 5:**
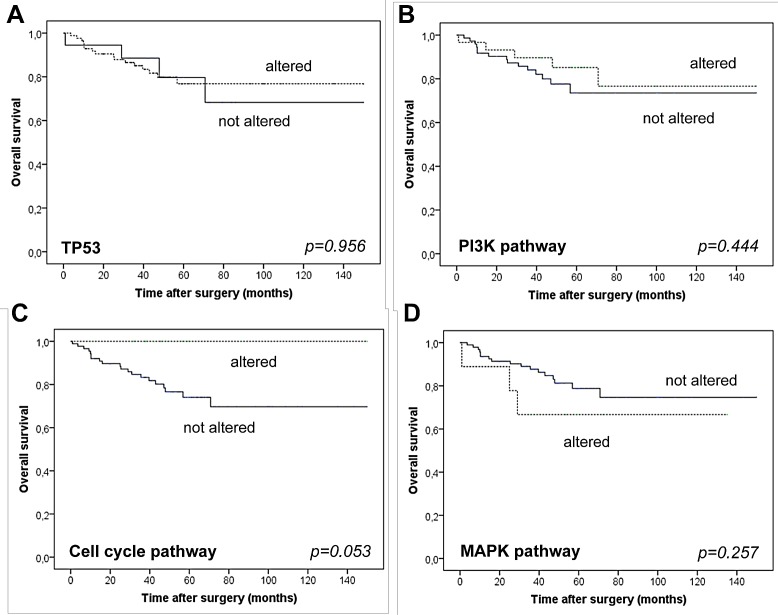
Overall survival of patients stratified for the presence/absence of mutations in *TP53* (A), the PI3K pathway (B), cell cycle pathways (C) and MAPK associated pathways (D).

Tumors harboring cell cycle alterations were associated with better overall survival (mean survival cell cycle wt: 50.7 months versus cell cycle mutant: not reached) but with no differences in disease free survival (Figure 5), however, the association was only of borderline significance (p=0.053). Strikingly, within the observation period none of the patients with a cell cycle mutation driven tumor had died. The other molecular groups were not associated with any differences in overall and disease free survival.

## DISCUSSION

TNBC are a heterogeneous group of tumors with different histological and molecular features. As in other tumor entities, it is not unlikely that molecular stratification of TNBC will become essential both in clinical and preclinical studies to determine the prognosis of patients and identify predictors for the response to differing treatment options. To build a basis for this likely development, using targeted ultradeep multigene parallel sequencing, we analyzed 104 formalin-fixed paraffin-embedded (FFPE) tissue samples from patients with clinically well characterized TNBC tumors in order to assess the validity of our technology, as well as to test for possible correlations between mutational profiles and morphology, immunohistochemistry as well as clinical outcome in TNBC.

The most prevalent molecular aberrations we identified were *TP53* mutations (82.7 %), followed by PIK3 pathway alterations (29.8 %), cell cycle- (14.4 %) and MAPK-pathway changes (8.7 %). This is well in line with data from WES studies using cryomaterial who found essentially the same groups of aberrations in this tumor entity [16].

It is well known that *TP53* mutations are found with high frequency in TNBC [15, 20], we detected *TP53* mutations in 83% of the cases in our cohort, mutations were usually observed with high allele frequencies. This supports the role of *TP53* mutations as the key genetic event in this population. However, in a small group of tumors (16.3 %) *TP53* mutated allele frequency suggested subclonality, consistent with a mutational event occurring later during tumor evolution [16]. This finding is well in accordance to previous studies [16].

In our study, PIK3 pathway alterations were found to be present in approximately 30% of TNBC. It is important to note that compared to previous WES studies [15-17, 21-23], we detected a considerably higher frequency of mutations involved in this pathway. The higher incidence of PI3K alterations in our cohort might be explained by the higher sequencing coverage of our targeted profiling approach when compared to WES studies. In the latter, specifically subclonal events might be missed. And indeed, the comparison of allele frequencies of mutations suggested that the most prevalent mutation in this group, which affected PIK3CA, was considerably more often subclonal than e.g.*TP53* mutations. Our data is supported by one recent study on TNBC, which also applied high coverage targeted multigene sequencing, in this study comparably high alteration rates in the PI3K pathway have been reported [14]. These results may have clinical implications in the near future, since recently developed PIK3 pathway inhibitors might become available for breast cancer treatment [24]. It is conceivable to speculate that differences in the activation state of the PI3K pathway might be associated with differing responses to the drugs in this setting.

Cell cycle regulators other than *TP53* have been found mutated in our cohort in 14% of the cases, with the most important gene affected being *RB*. This is perfectly in line with previous studies [15, 16]. Alterations in cell cycle regulators and especially in the RB pathway are a very relevant feature in TNBC [25, 26] and also include potential therapeutic targets [26].

The fourth group of alterations found by us comprised genes of the MAPK pathway. This mainly included *MAP2K4* amplifications and *HER2*-mutations. *MAP2K4* amplifications are well recognized in breast tumors, but until now were mainly found in the group of luminal mammary cancers [15]. In previous studies *HER2* mutations have been detected in around 3% of TNBC cases [16, 27]. From the therapeutic viewpoint, *Her2* mutations (3.8% of all cases in our study) in TNBC are of interest, since the fraction of *HER2* mutated TNBC might potentially be better included into the HER2-positive group of breast cancers. This is supported by the fact that activating *HER2* mutations in *HER2* gene amplification negative breast cancers have been successfully treated by anti-HER2 therapy [27].

The spectrum of potentially druggable targets in breast cancer is not limited to HER2 or hormone receptors and has risen over the last years [14, 16, 24, 28]. However, for TNBC, treatment regimens are still mainly based on the application of conventional chemotherapy and do not yet involve targeted approaches. Interestingly, in our cohort approximately 40% of the alterations identified can be targeted by drugs that are already in clinical use and when experimental drugs used in clinical trials are included, this number rises up to almost 90% [14]. However, the interplay between targeted drugs and the predictive value of molecular alterations is in most cases far from being fully understood [29] and it is well recognized that the percentage of detected potentially targetable molecular alterations does not directly mirror the potential treatment options. Therefore, it could certainly not be expected that we will be able to exploit all of these molecular alterations therapeutically.

Comparing our targeted multigene sequencing data with results from WES studies, we conclude that we can extract the same molecular profiles in a very time and cost effective manner. Due to the much higher read depth our approach also allows for a more sensitive detection of putatively subclonal genetic events. The precise definition of the percentage of subclonal events per driver mutation might substantially improve our understanding of TNBC tumor biology, however, to which extent subclonal events might also harbor a predictive value is entirely unknown at present.

On the other hand, our targeted multigene sequencing approach has some inherent limitations, which mainly reflect the tradeoff between cost effectiveness and comprehensiveness. Although we will detect some of the low frequency mutations in the “long tail” of the TNBC mutational landscape, by far not all of these alterations will be discovered since we are forced to restrict our analysis to a certain number of frequently altered genes. Also, very large genes without mutational hotspots, even though they are well established in breast tumorigenesis, such as *BRCA1* and *BRCA2*, are difficult to include in a cost-effective amplicon-based panel and have not been investigated in our approach. However, since sequencing costs are dropping [30], in the future we might extent the number of genes which can rationally be included in such a panel approach. This highlights once more the fact that targeted multigene panels will always represent a constant work in progress and must be adapted with maximal flexibility to the molecular as well as clinical data currently available.

Further, we noted an overlap of two or more genetic pathways to be altered in the same tumor in the majority of cases, which has also been found by others [14, 17]. This might have implications on the choice of therapy regimens, since it hints on a potential effectiveness of the combinatorial targeting of two mutated gene products, e.g. by blocking known cross talks between PIK3 signaling and TP53 [31] or MAPK signaling components [32-34].

Recently, we have published an immunohistochemistry (IHC) based study in which we have shown that by means of simple IHC four distinct clinically highly relevant subgroups of TNBC could be delineated, which were designated as luminal-like, basoluminal, basal A and basal B type, mainly based on the predominant pattern of cytokeratin expression [12]. The first group, luminal-like TNBC, is characterized by the lack of cytokeratin 5/6 and 14 as well as EGFR expression. We believe that this subtype represents de-differentiated luminal breast cancers, since BCL2 expression had been preserved in most cases, an anti-apoptotic protein frequently expressed in luminal A and luminal B breast cancer but generally not in HER2 positive or TNBC tumors [12, 35]. This assumption is supported by our sequencing data showing a comparably low frequency of *TP53* mutations in this tumor subgroup. The p53 pathway has been reported much less frequently altered in luminal tumors compared to the other subtypes [15]. This group of tumors also had the highest incidence of PI3K pathway alterations. Previously, it has been shown that BCL2 positive TNBC tend to be less responsive to anthracycline combination chemotherapy [36]. Therefore it is not surprising that these patients exhibit the worst overall survival in our previous study. However, the high incidence of PI3K pathway alterations might open the avenue of novel targeted treatment options in this patient population.

The second group of TNBC tumors delineated by IHC, also previously referred to as the “core basal phenotype” [37, 38], were characterized by EGFR overexpression, a moderate expression level of basal cytokeratins and a high expression of luminal cytokeratins. This subtype, which we have termed basoluminal, has been shown to correctly identify basal-like tumors as defined by gene expression studies with 100% specificity and 76% sensitivity [39]. In contrast, the third group, basal B tumors, lack cytoplasmic p16 accumulation and have a high Ki-67-index compared to luminal and basoluminal TNBC. With respect to mutational profiles both the basoluminal as well as the basal B phenotype were much alike. Both showed a comparable high rate of PI3K pathway alterations, which was in contrast to luminal-like tumors, accompanied by very high rates of *TP53* mutations in these tumors.

The fourth IHC group, basal A, was immunohistochemically characterized by accumulation of p16, overexpression of p53 and a high proliferation rate with Ki-67-indices over 70%. This association has been corroborated in several studies [40, 41] and overexpression of p16 has recently been linked to good response to adjuvant chemotherapy with significantly increased disease free survival [12, 40]. It has been speculated that the high Ki-67-index in this group is caused by inactivation of RB, thereby leading to an inactivation of the G_1_-S cell cycle checkpoint, and unblocked entry into the cell cycle [42]. However, this is not supported by our data, since we did not see relevant accumulations of cell cycle mutations in this subgroup. Although p53 overexpression detected by immunohistochemistry and *TP53* mutations do not have a perfect correlation [43], it has been shown that *TP53* mutations are usually accompanied by p53 overexpression and that the latter might be used as a marker for the presence of the former [43]. This is supported by our data since in the basal A TNBC subgroup usually expressing p53 we detected a very high percentage of *TP53* mutations (96%) and the overall correlation between the presence of *TP53* mutations and p53 protein expression was very tight.

Interestingly, although both basal TNBC subgroups are indistinguishable by histomorphology, in contrast to basal B tumors (35%), significantly less (4%) of the basal A tumors had PI3K pathway alterations, thereby providing evidence that both tumor groups harbor relevant discrepancies at the molecular level. The immunhistochemically defined basal A subtype might generally be a surrogate for a low level of *PIK3CA* mutations.

Besides immunohistochemical algorithms, gene expression analyses have been found useful for the stratification of TNBC into distinct molecular subgroups and have thereby prompted particular interest among scientists and researchers [11]. An overlay of expression profiling based TNBC subtypes and mutational profiles would clearly be of interest. However, expression profiling based TNBC subtyping traditionally has been done from frozen material, not from FFPE tissue and cryomaterial was not available for our cohort. Novel technologies such as Nanostring [44] might allow for reliable expression profiling from FFPE material but the question whether these datasets can be compared directly to data generated from cryomaterial is to some extent unanswered. We are currently working on this issue and might be able to address this topic for TNBC in the near future.

Comparing the histological subtypes invasive carcinoma NST, lobular carcinoma, medullary carcinoma and rare subtypes, we observed associations with certain mutations. Of note, we detected alterations in MAPK signaling only in invasive carcinoma NST but not in any other subtype. This is specifically interesting for *HER2* mutations since it might implicate that only this subtype must be tested for the presence of this alteration in the molecular diagnostic setting. However, sample size was quite low for lobular, medullary and rare carcinoma subtypes and therefore the results should be interpreted with caution and validated in larger cohorts of these rare tumors.

Interestingly, cell cycle alterations were associated with better overall survival but the association did only reach borderline statistical significance (p=0.053). An association with disease-free survival times was not observed. Although our data on this issue is, due to the low case number in the cell cycle group (n=15), somewhat premature, one may speculate that tumors with cell cycle activation might respond specifically well to conventional chemotherapy, which in general targets proliferating cells and is usually administered to patients with TNBC. Since differences in disease free survival were not present, the overall survival differences could not be related to differing responses to the initial adjuvant chemotherapeutic treatment. Yet, the overall survival differences may still be due to differing responses to chemotherapeutics administered later in the disease course once a recurrence has occurred. However, this clearly must be validated in future studies with higher case numbers.

Taken together we show that targeted breast cancer specific ultradeep multigene sequencing is a feasible, cost effective and time efficient method for a comprehensive molecular profiling of TNBC tumors. We could confirm the high frequency of potentially druggable molecular alterations in this tumor type with specifically high rates of PI3K pathway alterations, which clearly exceed the rates reported from exome sequencing programs. In addition, we showed that a simple IHC stratification approach might help to preselect molecularly defined TNBC subgroups and that certain molecular alterations might impact on patient survival in TNBC.

## MATERIAL AND METHODS

### Panel design

For the breast cancer panel design a large dataset of sequence variants in 778 breast cancers was downloaded from The Cancer Genome Atlas (TCGA) project [15]. The data were filtered for somatic mutations, being non-silent in the coding region resulting in 38.692 non-overlapping mutated genomic loci. Outlying expression coincident with copy number alterations (CNA) in 997 breast cancers was obtained from the METABRIC project. In the TCGA data, 56 loci were mutated in at least 1% and 437 loci were mutated in at least 0.5% of the 778 tumors available for analysis at this timepoint. We selected 112 loci that were (i) mutated in at least 1% of the 778 tumors or (ii) mutated in at least 0.5% of the 778 tumors and were located in 57 genes that were found frequently mutated in breast cancer or molecular subtypes of breast cancer (specifically TNBC) before. From the METABRIC data, we selected seven genes where overexpression was coincident with gene amplification and which were frequently altered in breast cancer: ERBB2, CCND1, ZNF703, PAK1, MYC, MDM2, RPS6KA1. Using the mutated loci and the amplified genes as input, we constructed a custom sequencing panel using the Ion AmpliSeq Designer (Life Technologies, Darmstadt, Germany) which ultimately included 137 Amplicons that were located in 44 genes (Table 2).

**Table 2:** Genes and exons included in our breast cancer panel Genes are printed in bold, exons included are given below each gene.

Genes in Breast Cancer Panel				
**AFF2** 2	**CDKN1B** 1	**KRAS** 2,3	**PIK3R1** 10,11,13	**TLR4** 4
**AKT1** 3	**CDKNA2** 1,2	**MAP2K4** 3,5,7-9	**PTEN** 1,3,5-8	**TP53** 4-10
**APC** 16	**CEP164** 13	**MAP3K1** 4,9,10,13,14,17-20	**PTPRD** 18,23,34	**USP36** 17
**ARID1A** 20	**CTCF** 3,4	**MDM2** 4,11	**RB1** 2,3,6,13,16-18,20-23	**ZNF703** 1
**BRAF** 15	**EGFR** 18-21	**MLL3** 7,14,23,43	**RBMX** 4	
***CASP8*** 3,9	**ERBB2** 19-21	**MYC** 2,3	**RPS6KA1** 11,14	
**CBFB** 3,4	**FGFR1** 5,14	**NOTCH1** 34	**RUNX1** 5,7,8	
**CCND1** 1,3	**GATA3** 5,6	**NR1H2** 6	**SF3B1** 14,15	
**CDH1** 2-7, 9-14, 16	**GIGYF2** 29	**PAK1** 2,13	**TBL1XR1** 5	
**CDK4** 4,7	**HERC1** 27	**PIK3CA** 2,5,8,10,14,21	**TBX3** 1,2	

### Cohort

Our tissue cohort consisted of 104 cases of TNBC samples. Negative estrogen- and progesteron receptor status of the tumors was defined by positivity in <1% of tumor cells according to ASCO/CAP Guidelines [45]. HER2 negativity was defined by absence of membranous staining or weak discontinuous membranous staining. Cases with moderate membranous staining in >10% of tumor cells were examined by in-situ hybridization according to ASCO/CAP Guidelines [46] and only negative cases were included. The samples were provided by the tissue bank of the National Center for Tumor Diseases (NCT, Heidelberg, Germany) in accordance with the local regulations and the approval of the ethics committee of the University of Heidelberg. All patients were diagnosed and treated between 2003 and 2006. 19 patients (18.3%) received neoadjuvant chemotherapy. Median age was 52.0 years (range: 28-90). Clinicopathological characteristics of the cohort are given in Table 1. Overall survival (OS) data was present for all but one patient, data on disease-free survival was present for 98 patients, patients still alive after 150 months were censored at this timepoint for OS analyses. 20 patients (19.2%) died during follow up, 51 (49%) relapsed. Median follow-up time of patients still alive at the endpoint of analysis was 59.4 months (range: 10-150 months), median follow-up time of patients without relapse at the endpoint of analysis was 46.8 months (range: 11-135).

### DNA preparation

Tumor areas were marked on an H&E stained slide and corresponding tissue areas were microdissected from three subsequent unstained slides. Tumor cell content was documented, for later correction of allele frequencies. Extraction of genomic DNA was performed by proteinase K digestion and fully automated purification using either the QIA Symphony SP (Qiagen, Hilden, Germany) or the Maxwell 16 Research System (Promega, Madison, USA). DNA content was measured fluorimetrically using the QuBit HS DNA Assay (Life Technologies) and DNA sequencing grade quality was confirmed using a real-time qPCR-based method (RNAseP Detection system, Life Technologies).

### Library preparation and semiconductor sequencing

For library preparation, the multiplex PCR-based Ion Torrent AmpliSeq^TM^ technology (Life Technologies) with a custom made Breast Cancer Panel was used (see above). Amplicon library preparation was performed with the Ion AmpliSeq Library Kit v2.0 using 10ng of DNA. Briefly, the DNA was mixed with the primer pool, containing all primers for generating the 137 amplicons and the AmpliSeq HiFi Master Mix and transferred to a PCR cycler (BioRad, Munich, Germany). After the end of the PCR reaction, primer end sequences were partially digested using FuPa reagent, followed by the ligation of barcoded sequencing adapters (Ion Xpress Barcode Adapters 1-16, Life Technologies). The final library was purified using AMPure XP magnetic beads (Beckman Coulter, Krefeld, Germany) and quantified using qPCR (Ion Library Quantitation Kit, Life Technologies) on a StepOne qPCR machine (Life Technologies). The individual libraries were diluted to a final concentration of 100pM and eight to ten libraries were pooled and processed to library amplification on Ion Spheres using Ion PGM™ Template OT2 200 Kit. Unenriched libraries were quality-controlled using Ion Sphere quality control measurement on a QuBit instrument. After library enrichment (Ion OneTouch ES), the library was processed for sequencing using the Ion Torrent 200bp sequencing v2 chemistry and the barcoded libraries were loaded onto a chip. Our way of pooling eight samples on a 318 chip resulted in a mean coverage of approximately 3000 fold per amplicon.

### Variant Calling and Annotation

Data analyses were performed using the Ion Torrent Suite Software (version 3.6). After base calling, the reads were aligned against the human genome (hg19) using the TMAP algorithm within the Torrent Suite. Variant calling was performed with the variant caller plugin within the Torrent Suite Software using a corresponding bed-file containing the coordinates of the amplified regions. Only variants with an allele frequency > 5% and minimum coverage > 200 reads were taken into account. Variant annotation was performed using the CLC Genomics Workbench (version 6.5). Annotations included information about nucleotide and amino acid changes of RefSeq annotated genes, COSMIC and dbSNP entries as well as detection of possible splice site mutations. For data interpretation and verification, the aligned reads were visualized using the IGV browser (Broad Institute) [47]. For further single nucleotide polymorphisms (SNP) analysis, only non-synonymous nucleotide exchanges were considered. Each SNP was compared to entries in the COSMIC, dbSNP and 5000 Exomes databases. If a SNP was annotated as being of germline origin in the dbSNP database and was not annotated as a mutation in COSMIC and PubMED, this SNP was disregarded. SNPs without any annotation were initially included. However, those SNPs without annotation for which the raw allele frequency (not corrected for tumor cell content) observed approached 100%, were disregarded since we assume that these alterations were of germline origin, as well.

### Copy number variations

To enable CNV detection using targeted multigene sequencing, we made use of the cn.MOPS algorithm, as implemented in the Bioconductor package of the same name. The cn.MOPS software [48] does not rely on the presence of normal-tissue controls. It accurately detects CNVs as a read - depth variation along multiple cancer samples. To this end, the genome is segmented into the available amplicons of the breast cancer panel. For each of these predefined segments a local parameter estimation of a mixed Poisson distribution is performed. Using a Bayesian approach, cn.MOPS decomposes variations in the depth of coverage across samples into integer copy numbers and noise by means of its mixture components and the estimated Poisson distributions, respectively. This allows us to filter for detections, which are caused by experimental noise, and to determine the copy number of each specific segment. A gene will be considered amplified if more than half of the exons covered by the applied amplicon panel showed a statistically valid change in their copy number status.

### Statistical analysis

Statistical analyses were carried out with SPSS 20 (IBM, Armonk, USA) and GraphPad Prism 4 (GraphPad Software, La Jolla, USA). The significance of correlations between molecular groups and clinico-pathological data were tested by Fisher’s exact test, χ2 test for trends and unpaired t-test as indicated. Survivor curves were estimated by the Kaplan-Meier method. Differences in survival were assessed by the log rank test. P-values <0.05 were considered statistical significant.

## References

[1] WHO (2014). World Health Organization: Breast Cancer burden. http://www.who.int/cancer/detection/breastcancer/en/index1.html.

[2] Perou CM, Sorlie T, Eisen MB, van de Rijn M, Jeffrey SS, Rees CA, Pollack JR, Ross DT, Johnsen H, Akslen LA, Fluge O, Pergamenschikov A, Williams C, Zhu SX, Lonning PE, Borresen-Dale AL (2000). Molecular portraits of human breast tumours. Nature.

[3] Sorlie T, Wang Y, Xiao C, Johnsen H, Naume B, Samaha RR, Borresen-Dale AL (2006). Distinct molecular mechanisms underlying clinically relevant subtypes of breast cancer: gene expression analyses across three different platforms. BMC genomics.

[4] Paik S, Kim C, Wolmark N (2008). HER2 status and benefit from adjuvant trastuzumab in breast cancer. The New England journal of medicine.

[5] Goldhirsch A, Winer EP, Coates AS, Gelber RD, Piccart-Gebhart M, Thurlimann B, Senn HJ, Panel m (2013). Personalizing the treatment of women with early breast cancer: highlights of the St Gallen International Expert Consensus on the Primary Therapy of Early Breast Cancer 2013. Annals of oncology: official journal of the European Society for Medical Oncology / ESMO.

[6] Foulkes WD, Smith IE, Reis-Filho JS (2010). Triple-negative breast cancer. The New England journal of medicine.

[7] Farmer H, McCabe N, Lord CJ, Tutt AN, Johnson DA, Richardson TB, Santarosa M, Dillon KJ, Hickson I, Knights C, Martin NM, Jackson SP, Smith GC, Ashworth A (2005). Targeting the DNA repair defect in BRCA mutant cells as a therapeutic strategy. Nature.

[8] Sanga S, Broom BM, Cristini V, Edgerton ME (2009). Gene expression meta-analysis supports existence of molecular apocrine breast cancer with a role for androgen receptor and implies interactions with ErbB family. BMC medical genomics.

[9] Prat A, Parker JS, Karginova O, Fan C, Livasy C, Herschkowitz JI, He X, Perou CM (2010). Phenotypic and molecular characterization of the claudin-low intrinsic subtype of breast cancer. Breast cancer research: BCR.

[10] Kreike B, van Kouwenhove M, Horlings H, Weigelt B, Peterse H, Bartelink H, van de Vijver MJ (2007). Gene expression profiling and histopathological characterization of triple-negative/basal-like breast carcinomas. Breast cancer research: BCR.

[11] Lehmann BD, Bauer JA, Chen X, Sanders ME, Chakravarthy AB, Shyr Y, Pietenpol JA (2011). Identification of human triple-negative breast cancer subtypes and preclinical models for selection of targeted therapies. The Journal of clinical investigation.

[12] Elsawaf Z, Sinn HP, Rom J, Bermejo JL, Schneeweiss A, Aulmann S (2013). Biological subtypes of triple-negative breast cancer are associated with distinct morphological changes and clinical behaviour. Breast.

[13] Poumpouridou N, Kroupis C (2012). Hereditary breast cancer: beyond BRCA genetic analysis; PALB2 emerges. Clinical chemistry and laboratory medicine: CCLM / FESCC.

[14] Balko JM, Giltnane JM, Wang K, Schwarz LJ, Young CD, Cook RS, Owens P, Sanders ME, Kuba MG, Sanchez V, Kurupi R, Moore PD, Pinto JA, Doimi FD, Gomez H, Horiuchi D (2014). Molecular profiling of the residual disease of triple-negative breast cancers after neoadjuvant chemotherapy identifies actionable therapeutic targets. Cancer discovery.

[15] Cancer Genome Atlas N (2012). Comprehensive molecular portraits of human breast tumours. Nature.

[16] Shah SP, Roth A, Goya R, Oloumi A, Ha G, Zhao Y, Turashvili G, Ding J, Tse K, Haffari G, Bashashati A, Prentice LM, Khattra J, Burleigh A, Yap D, Bernard V (2012). The clonal and mutational evolution spectrum of primary triple-negative breast cancers. Nature.

[17] Stephens PJ, Tarpey PS, Davies H, Van Loo P, Greenman C, Wedge DC, Nik-Zainal S, Martin S, Varela I, Bignell GR, Yates LR, Papaemmanuil E, Beare D, Butler A, Cheverton A, Gamble J (2012). The landscape of cancer genes and mutational processes in breast cancer. Nature.

[18] Salto-Tellez M, de Castro DG (2014). Next Generation Sequencing: A Change of Paradigm in Molecular Diagnostic Validation. The Journal of pathology.

[19] Endris V, Penzel R, Warth A, Muckenhuber A, Schirmacher P, Stenzinger A, Weichert W (2013). Molecular diagnostic profiling of lung cancer specimens with a semiconductor-based massive parallel sequencing approach: feasibility, costs, and performance compared with conventional sequencing. The Journal of molecular diagnostics: JMD.

[20] Turner N, Moretti E, Siclari O, Migliaccio I, Santarpia L, D’Incalci M, Piccolo S, Veronesi A, Zambelli A, Del Sal G, Di Leo A (2013). Targeting triple negative breast cancer: is p53 the answer?. Cancer treatment reviews.

[21] Tilch E, Seidens T, Cocciardi S, Reid LE, Byrne D, Simpson PT, Vargas AC, Cummings MC, Fox SB, Lakhani SR, Chenevix Trench G (2014). Mutations in EGFR, BRAF and RAS are rare in triple-negative and basal-like breast cancers from Caucasian women. Breast cancer research and treatment.

[22] Engebraaten O, Vollan HK, Borresen-Dale AL (2013). Triple-negative breast cancer and the need for new therapeutic targets. The American journal of pathology.

[23] Santarpia L, Qi Y, Stemke-Hale K, Wang B, Young EJ, Booser DJ, Holmes FA, O’Shaughnessy J, Hellerstedt B, Pippen J, Vidaurre T, Gomez H, Valero V, Hortobagyi GN, Symmans WF, Bottai G (2012). Mutation profiling identifies numerous rare drug targets and distinct mutation patterns in different clinical subtypes of breast cancers. Breast cancer research and treatment.

[24] O’Toole SA, Beith JM, Millar EK, West R, McLean A, Cazet A, Swarbrick A, Oakes SR (2013). Therapeutic targets in triple negative breast cancer. Journal of clinical pathology.

[25] Curtis C, Shah SP, Chin SF, Turashvili G, Rueda OM, Dunning MJ, Speed D, Lynch AG, Samarajiwa S, Yuan Y, Graf S, Ha G, Haffari G, Bashashati A, Russell R, McKinney S (2012). The genomic and transcriptomic architecture of 2,000 breast tumours reveals novel subgroups. Nature.

[26] Speers C, Tsimelzon A, Sexton K, Herrick AM, Gutierrez C, Culhane A, Quackenbush J, Hilsenbeck S, Chang J, Brown P (2009). Identification of novel kinase targets for the treatment of estrogen receptor-negative breast cancer. Clinical cancer research: an official journal of the American Association for Cancer Research.

[27] Bose R, Kavuri SM, Searleman AC, Shen W, Shen D, Koboldt DC, Monsey J, Goel N, Aronson AB, Li S, Ma CX, Ding L, Mardis ER, Ellis MJ (2013). Activating HER2 mutations in HER2 gene amplification negative breast cancer. Cancer discovery.

[28] Baselga J (2011). Targeting the phosphoinositide-3 (PI3) kinase pathway in breast cancer. The oncologist.

[29] Andre F, O’Regan R, Ozguroglu M, Toi M, Xu B, Jerusalem G, Masuda N, Wilks S, Arena F, Isaacs C, Yap YS, Papai Z, Lang I, Armstrong A, Lerzo G, White M (2014). Everolimus for women with trastuzumab-resistant, HER2-positive, advanced breast cancer (BOLERO-3): a randomised, double-blind, placebo-controlled phase 3 trial. The lancet oncology.

[30] MacConaill LE (2013). Existing and emerging technologies for tumor genomic profiling. Journal of clinical oncology: official journal of the American Society of Clinical Oncology.

[31] Adams JR, Xu K, Liu JC, Agamez NM, Loch AJ, Wong RG, Wang W, Wright KL, Lane TF, Zacksenhaus E, Egan SE (2011). Cooperation between Pik3ca and p53 mutations in mouse mammary tumor formation. Cancer research.

[32] Saini KS, Loi S, de Azambuja E, Metzger-Filho O, Saini ML, Ignatiadis M, Dancey JE, Piccart-Gebhart MJ (2013). Targeting the PI3K/AKT/mTOR and Raf/MEK/ERK pathways in the treatment of breast cancer. Cancer treatment reviews.

[33] De Luca A, Maiello MR, D’Alessio A, Pergameno M, Normanno N (2012). The RAS/RAF/MEK/ERK and the PI3K/AKT signalling pathways: role in cancer pathogenesis and implications for therapeutic approaches. Expert opinion on therapeutic targets.

[34] Craig DW, O’Shaughnessy JA, Kiefer JA, Aldrich J, Sinari S, Moses TM, Wong S, Dinh J, Christoforides A, Blum JL, Aitelli CL, Osborne CR, Izatt T, Kurdoglu A, Baker A, Koeman J (2013). Genome and transcriptome sequencing in prospective metastatic triple-negative breast cancer uncovers therapeutic vulnerabilities. Molecular cancer therapeutics.

[35] Kolacinska A, Chalubinska J, Zawlik I, Szymanska B, Borowska-Garganisz E, Nowik M, Fendler W, Kubiak R, Pawlowska Z, Morawiec Z, Szemraj J (2012). Apoptosis-, proliferation, immune function-, and drug resistance- related genes in ER positive, HER2 positive and triple negative breast cancer. Neoplasma.

[36] Abdel-Fatah TM, Perry C, Dickinson P, Ball G, Moseley P, Madhusudan S, Ellis IO, Chan SY (2013). Bcl2 is an independent prognostic marker of triple negative breast cancer (TNBC) and predicts response to anthracycline combination (ATC) chemotherapy (CT) in adjuvant and neoadjuvant settings. Annals of oncology: official journal of the European Society for Medical Oncology / ESMO.

[37] Tischkowitz M, Brunet JS, Begin LR, Huntsman DG, Cheang MC, Akslen LA, Nielsen TO, Foulkes WD (2007). Use of immunohistochemical markers can refine prognosis in triple negative breast cancer. BMC cancer.

[38] Cheang MC, Voduc D, Bajdik C, Leung S, McKinney S, Chia SK, Perou CM, Nielsen TO (2008). Basal-like breast cancer defined by five biomarkers has superior prognostic value than triple-negative phenotype. Clinical cancer research: an official journal of the American Association for Cancer Research.

[39] Nielsen TO, Hsu FD, Jensen K, Cheang M, Karaca G, Hu Z, Hernandez-Boussard T, Livasy C, Cowan D, Dressler L, Akslen LA, Ragaz J, Gown AM, Gilks CB, van de Rijn M, Perou CM (2004). Immunohistochemical and clinical characterization of the basal-like subtype of invasive breast carcinoma. Clinical cancer research: an official journal of the American Association for Cancer Research.

[40] Bogina GS, Lunardi G, Marcolini L, Brunelli M, Bortesi L, Marconi M, Coati F, Valerio M, Guerriero M, Massocco A, Pegoraro MC, Zamboni G (2014). P16 but not retinoblastoma expression is related to clinical outcome in no-special-type triple-negative breast carcinomas. Modern pathology: an official journal of the United States and Canadian Academy of Pathology, Inc.

[41] Shan M, Zhang X, Liu X, Qin Y, Liu T, Liu Y, Wang J, Zhong Z, Zhang Y, Geng J, Pang D (2013). P16 and p53 play distinct roles in different subtypes of breast cancer. PloS one.

[42] Subhawong AP, Subhawong T, Nassar H, Kouprina N, Begum S, Vang R, Westra WH, Argani P (2009). Most basal-like breast carcinomas demonstrate the same Rb-/p16+ immunophenotype as the HPV-related poorly differentiated squamous cell carcinomas which they resemble morphologically. The American journal of surgical pathology.

[43] Boyle DP, McArt DG, Irwin G, Wilhelm-Benartzi CS, Lioe TF, Sebastian E, McQuaid S, Hamilton PW, James JA, Mullan PB, Catherwood MA, Harkin DP, Salto-Tellez M (2014). The prognostic significance of the aberrant extremes of p53 immunophenotypes in breast cancer. Histopathology.

[44] Filipits M, Nielsen TO, Rudas M, Greil R, Stoger H, Jakesz R, Bago-Horvath Z, Dietze O, Regitnig P, Gruber-Rossipal C, Muller-Holzner E, Singer CF, Mlineritsch B, Dubsky P, Bauernhofer T, Hubalek M (2014). The PAM50 risk-of-recurrence score predicts risk for late distant recurrence after endocrine therapy in postmenopausal women with endocrine-responsive early breast cancer. Clinical cancer research: an official journal of the American Association for Cancer Research.

[45] Hammond ME, Hayes DF, Dowsett M, Allred DC, Hagerty KL, Badve S, Fitzgibbons PL, Francis G, Goldstein NS, Hayes M, Hicks DG, Lester S, Love R, Mangu PB, McShane L, Miller K (2010). American Society of Clinical Oncology/College of American Pathologists guideline recommendations for immunohistochemical testing of estrogen and progesterone receptors in breast cancer (unabridged version). Archives of pathology & laboratory medicine.

[46] Wolff AC, Hammond ME, Hicks DG, Dowsett M, McShane LM, Allison KH, Allred DC, Bartlett JM, Bilous M, Fitzgibbons P, Hanna W, Jenkins RB, Mangu PB, Paik S, Perez EA, Press MF (2013). Recommendations for human epidermal growth factor receptor 2 testing in breast cancer: American Society of Clinical Oncology/College of American Pathologists clinical practice guideline update. Journal of clinical oncology: official journal of the American Society of Clinical Oncology.

[47] Robinson JT, Thorvaldsdottir H, Winckler W, Guttman M, Lander ES, Getz G, Mesirov JP (2011). Integrative genomics viewer. Nature biotechnology.

[48] Klambauer G, Schwarzbauer K, Mayr A, Clevert DA, Mitterecker A, Bodenhofer U, Hochreiter S (2012). cn. MOPS: mixture of Poissons for discovering copy number variations in next-generation sequencing data with a low false discovery rate. Nucleic acids research.

